# Genotyping of *Giardia duodenalis* in children in upper Egypt using assemblage- specific PCR technique

**DOI:** 10.1371/journal.pone.0240119

**Published:** 2020-10-01

**Authors:** Alzahraa Abdelraouf Ahmad, Asmaa M. El-Kady, Tasneem M. Hassan

**Affiliations:** 1 Department of Medical Parasitology, Faculty of Medicine, Assiut University, Assiut, Egypt; 2 Department of Medical Parasitology, Faculty of Medicine, South Valley University, Qena, Egypt; Beni Suef University, Faculty of Veterinary Medicine, EGYPT

## Abstract

*Giardia duodenalis* is a common gastrointestinal protozoan parasite, causing diarrheal illness in humans worldwide. Yet, the distribution of *G*. *duodenalis* genotypes among human patients and their clinical relevance remains controversial. This study aimed to detect *G*. *duodenalis* in children in Upper Egypt and identify causative genotypes and elucidate a possible correlation between genotype and clinical presentation. One hundred sixty-five children, regardless of symptoms, were tested for giardiasis. *Giardia* positive stool samples (40/165) were subjected to PCR amplification targeting the *tpi* gene with positive PCR results in only 35 cases (87.5%). Assemblage-specific amplification of genotypes (A, B, and the zoonotic E strains) revealed predominantly *G*. *duodenalis* Assemblage A (45.7%). Assemblage B and mixed A and B infections were detected in 31.4% and 22.8% of children, respectively. Assemblage E was not detected. *G*. *duodenalis* assemblage A was dominant in children who complained of diarrhea and abdominal cramps. In contrast, asymptomatic children with positive stool samples display a higher frequency of assemblage B and mixed infections. The study highlights the predominance of *Giardia* Assemblage A in our study locality. This study is the first for this endemic area to use the copro-PCR technique for diagnosis and genotyping of giardiasis. Study results show the value of simple species-specific primers for genotyping in communities with little access to laboratory resources. Further genetic studies are needed to clarify the association between parasite genetic diversity and patient symptomatology.

## Introduction

*Giardia duodenalis* is a cosmopolitan protozoan parasite that affects a wide range of vertebrates, including humans. The WHO included giardiasis in its ‘Neglected Diseases Initiative’ in 2004 in recognition of its significant socio-economic impacts [1]. *Giardia duodenalis* is a common gastrointestinal pathogen that induces diarrhea, particularly in children in low-income countries [2–4]. It has been also associated with impaired growth and cognitive function in poor resource settings [5–7] and developed countries [8]. Human giardiasis prevalence in developed nations ranges from 1 to 8% of the population; however, in developing countries, it exceeds 30% of the population [9]. The prevalence rate in Egypt is up to 30.2% [4].

The distribution of *G*. *duodenalis* among humans varies widely as do clinical presentations, which range from absence of gastrointestinal symptoms to acute symptoms of diarrhea, abdominal pain, flatulence, nausea. Persistent infection can lead to chronic diarrhea, weight loss, and even malabsorption that may cause serious effects on growth and intellectual development of children [10–12]

Populations most affected by giardiasis are immunocompromised persons and travelers to areas with high endemic infection rates [13]. However, children are the high-risk group for giardiasis, especially developing countries with inadequate sanitation [1].

The main routes for exposure of infective *G*. *duodenalis* cysts are fecal-oral transmission through contaminated food or water sources and direct contact with infected humans or animals [14]. Thus, zoonotic transmission is of great concern [15].

Giardiasis is a challenging disease with increasing spread in the environment. However, routine diagnostic methods still lack sensitivity. Molecular analysis for diagnosis of human pathogens using copro-PCR is a valuable tool with acceptable sensitivity for detection of *G*. *duodenalis* in human stools [2].

Molecular characterization of *G*. *duodenalis* from different localities reflects complex genetic profiles. Analysis using different molecular tools have targeted several genes, including small-subunit ribosomal RNA (*ssu-rRNA*), β-giardin (*bg*), glutamate dehydrogenase (*gdh*), and triose-phosphate isomerase(*tpi*) genes [16]. *Giardia duodenalis* comprises eight major genotypes (Assemblages A to H) based on these loci [17]. The most frequently isolated *G*. *duodenalis* assemblages from human stool samples are A and B [18], which are also found in several other mammalian hosts [14]. Other assemblages (C, E, and F) are sporadically isolated from humans in some parts of Africa including Egypt [19, 20]. Assemblage B is responsible for most human infections (58% of the cases). Assemblage A accounts for 37% [17].

To date, the relationship between *G*. *duodenalis* genotypes and clinical presentation is still unclear. Published studies have reported conflicting results. Some reported a strong correlation between assemblages and clinical symptoms while others found no such relationship [21–25]. Large epidemiological case-control studies conducted in African countries have demonstrated that *Giardia duodenalis* infections do not seem to be positively associated with acute diarrhoea in young children [26–29]. The present study was designed to update genotyping of *G*. *duodenalis* in children in Upper Egypt and examine possible correlations among detected genotypes and clinical presentations.

## Materials and methods

### Study type and populations

The current cross-sectional study was performed during the period from March to May 2018. Participants were 165 children aged three to 12 who visited the outpatient clinic at the Assiut University Children’s Hospital, Egypt, to seek medical advice. Children complained of a range of gastrointestinal symptoms, such as diarrhea, flatulence, and abdominal cramps, and weight loss. Some children were asymptomatic, and stool samples were collected as a part of routine investigations.

### Ethical consideration

Informed consent was obtained from children’s guardians and the research was approved by the Ethical Review Board of the Faculty of Medicine, Assiut University. Children positive for any parasitic infection were treated based on clinical presentation and findings in the Pediatric Department.

### Collection and processing of samples

A stool sample from each participant was obtained in a dry, clean, labeled plastic container. Each stool sample was divided into three portions. The first portion was used for direct smear examination with Lugol’s iodine, the second was preserved in formalin-saline fixative for concentration and microscopic analysis, and the third was stored without the addition of preservative at—20 °C in Eppendorf tubes for molecular studies.

### Copro-nPCR assays

#### Genomic DNA extraction from stool samples

Stool samples microscopically positive for *Giardia* were submitted for DNA extraction in the Molecular Laboratory of the Center of Excellence for Medical Research, Faculty of Medicine, Assiut University using a QIAamp^®^ DNA Stool Mini Kit (cat. no. 51504). Extraction of genomic DNA followed the manufacturer’s instructions with some modification. Incubation time and temperature were one hour at 95°C and elution of the extracted DNA used 100 μl of elution buffer. The concentration and purity of DNA were characterized (Qubit^®^ 2.0 Fluorometer) and preserved at—20 °C until use in PCR analysis.

#### Amplification of the *tpi* gene of *Giardia duodenalis*

PCR amplification of the *tpi* gene for molecular identification of *G*. *duodenalis* was performed as described by Sulaiman et al. [30] to generate a PCR product (605 bp) using the following primer sets; AL3543 as the forward primer and AL3546 as the reverse primer (Table 1).

**Table 1:** The sequence of the primers used in the present study.

Primer	Sequence	Accession No.	Size	Reference
*tpi* gene	AL3543 5′-AAATIATGCCTGCTCGTCG-3′	U57897, AF06957 to AF069563, L02116, L02120	605 bp	[30]
	AL3546 5′-CAAACCTTITCCGCAAACC-3′			
Assemblage A	Af: 5′-CGC CGT ACA CCT GTC A-3′	AY368157 to AY368161, GIU57897, and AY655704,	332 bp	[31]
	Ar: 5′-AGC AAT GAC AAC CTC CTT CC-3′			
Assemblage B	AssBF: 5′ GTT GTT GTT GCT CCC TCC TTT 3′	AY228628 AY228632, AF069560 and AY228634	400 bp	[32]
	AssBR: 5′ CCG GCT CAT AGG CAATTA CA 3′			
Assemblage E	Ef: 5′-CCC CTT CTG CCG TAC ATTTAT-3′	AY228645 to AY228647, and AY655705 to AY655706,	388 bp	[31]
	Er: 5′-GGC TCG TAA GCA ATA ACG ACT T-3′			

The reaction mixture used 25 μl total volume, including 2 μl DNA template, 12.5 μl PCR master mix (1 U of *Taq* polymerase, 250 μM each of deoxynucleoside triphosphate (dNTP), 10 mM Tris-HCl, 30 mM KCl, 1.5 mM MgCl_2_), 1 μl of each primer, and 8.5 μl nuclease-free water. PCR reaction conditions were: initial denaturation at 95°C for 5 min, 35 cycles of 95°C denaturation for 45 sec, annealing at 50°C for 45 sec and extension at 72°C for 60 sec, and final extension step 72°C for 5 min.

#### Assemblage-specific amplification of *Giardia duodenalis* genotypes

Nested PCR with assemblage-specific primers was used to detect Assemblages A, B, and the zoonotic E strain as previously described [31, 32]. A secondary reaction was performed separately for each assemblage using a 1/10 dilution of the first PCR product of the *tpi* gene as a template.

The reaction conditions were the same as the first run except that annealing temperature for primers A and B was 62 °C and 67 °C for E Primers. A Veriti ^™^ 96-well thermal cycler (9902, Singapore) was used for PCR amplification. Amplified PCR products were analyzed in 1.5% agarose gels stained with ethidium bromide using horizontal electrophoresis (Compact M, Biometra, Germany). DNA fragments were visualized under UV illumination. The size of DNA fragments was compared with a 100-bp DNA ladder (Thermo Scientific^™^, Waltham, Massachusetts, USA).

### Statistical analysis

The study results were analyzed using SPSS software version 16. Categorical and quantitative variables were expressed in percentages. Statistical significance analysis of categorical data used Pearson’s chi-squared and Fisher’s exact test. The calculated *p-*value < 0.05 was considered statistically significant.

## Results

Participants were children from three to 12 years old. Ninety-three were boys (56.4%), and 88 (53.3%) were from rural areas. Participants presented with a range of symptoms, 75 (44.5%) complained of diarrhea, 22 (13.3%) of flatulence, 19 (11.5%) of abdominal cramps, and 17 (10.3%) weight loss. The remaining 32 (19.4%) patients were asymptomatic, and stool samples were collected as part of routine investigations (Table 2).

**Table 2:** The sociodemographic data of patients participating in the present study.

Residence	Symptomatology	Gender		Total (n./%)
		Female	Male	(No. = 165)
**Rural**	**Abdominal cramps**	0	5	5/88 (5.6)
	**Asymptomatic**	8	9	17/88 (19.3)
	**Diarrhea**	23	26	49/88 (55.7)
	**Flatulence**	8	4	12/88 (13.6)
	**Weight loss**	1	4	5/88 (5.6)
**Total**		**40 (24.2)**	**48 (29.1)**	**88 (53.3)**
**Urban**	**Abdominal cramps**	7	7	14/77 (18.2)
	**Asymptomatic**	4	11	15/77 (19.5)
	**Diarrhea**	11	15	26/77 (33.8)
	**Flatulence**	6	4	10/77(12.9)
	**Weight loss**	4	8	12/77 (15.6)
**Total**		**32 (19.4)**	**45 (27.3)**	**77 (46.7)**

**Fig 1:**
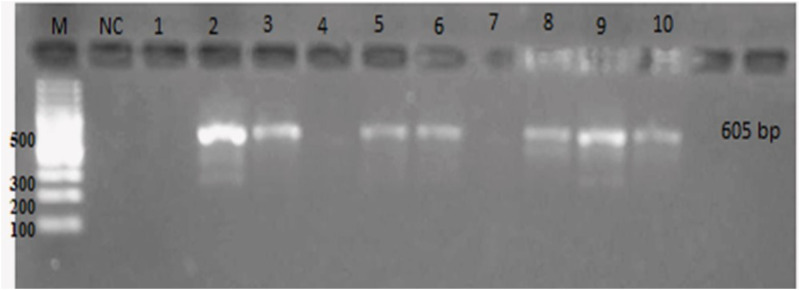
Agarose gel 1.5% stained with ethidium bromide showing PCR products of *tpi* gene amplification of *Giardia duodenalis*. Lane M: Molecular weight marker (100 bp), lane NC: negative control. Lanes (1 to 10): patient samples. The lanes with positive PCR products at 605 bp. Lanes (1, 4 & 7): negative samples.

Microscopic examination for *G*. *duodenalis* identified 40 (24.2%) of 165 cases as positive for giardiasis infection (Table 3). The majority were males (23/40) (57.5%). Children positive for infection were predominantly from rural areas (24/40) (60%). Also, the estimated prevalence of *Giardia* infection among symptomatic and asymptomatic cases showed a significant association with male patients (Table 4). However, molecular detection for *G*. *duodenalis* identified a positive *tpi* gene band of 605 bp in only 35 cases (87.5%) (Fig 1).

**Table 3:** The prevalence of *Giardia* infection among patients with different symptoms.

Patient complaints	Microscopic Results		Total (No./%)	*P*. value
	Negative for *Giardia* (No./%)	Positive for *Giardia* (No./%)		
**Abdominal cramps**	**14/125 (11.2)**	**5/40 (12.5)**	**19/165 (11.5)**	0.019**
**Diarrhea**	**62/125 (49.6)**	**13/40 (32.5)**	**75/165 (45.5)**	
**Flatulence**	**18/125 (14.4)**	**4/40 (10)**	**22/165 (13.3)**	
**Weight loss**	**14/125 (11.2)**	**3/40 (7.5)**	**17/165 (10.3)**	
**Asymptomatic**	**17/125 (13.6)**	**15/40 (37.5)**	**32/165 (19.4)**	
**Total**	**125 (75.75)**	**40(24.24)**	**165 (100)**	

**p-value: < 0.05 is considered statistically significant.

**Table 4:** The prevalence of *Giardia* infection among symptomatic and asymptomatic cases in relation to gender.

Gender			*Giardia* Microscopic Result		Total (No./%)	*P* value
			Negative (total no. = 125)	Positive (total no. = 40)		
**Female**	**Complaint**	**Abdominal cramps**	7	0	7/72 (9.7)	
		**Diarrhea**	25	9	34/72 (47.2)	
		**Flatulence**	11	3	14/72 (19.4)	
		**Weight loss**	5	0	5/72 (6.9)	0.073
		**Asymptomatic**	7	5	12/72 (16.7)	
	**Total**		55/125 (44)	17/40 (42.5)	72 (100)	
**Male**	**Complaint**	**Abdominal cramps**	7	5	12/93 (12.9)	
		**Diarrhea**	37	4	41/93 (44.1)	
		**Flatulence**	7	1	8/93 (8.6)	0.006**
		**Weight loss**	9	3	12/93 (12.9)	
		**Asymptomatic**	10	10	20/93 (21.5)	
	**Total**		70/125 (56)	23/40 (57.5)	93(100)	

**p-value: < 0.05 is considered statistically significant.

**Fig 2:**
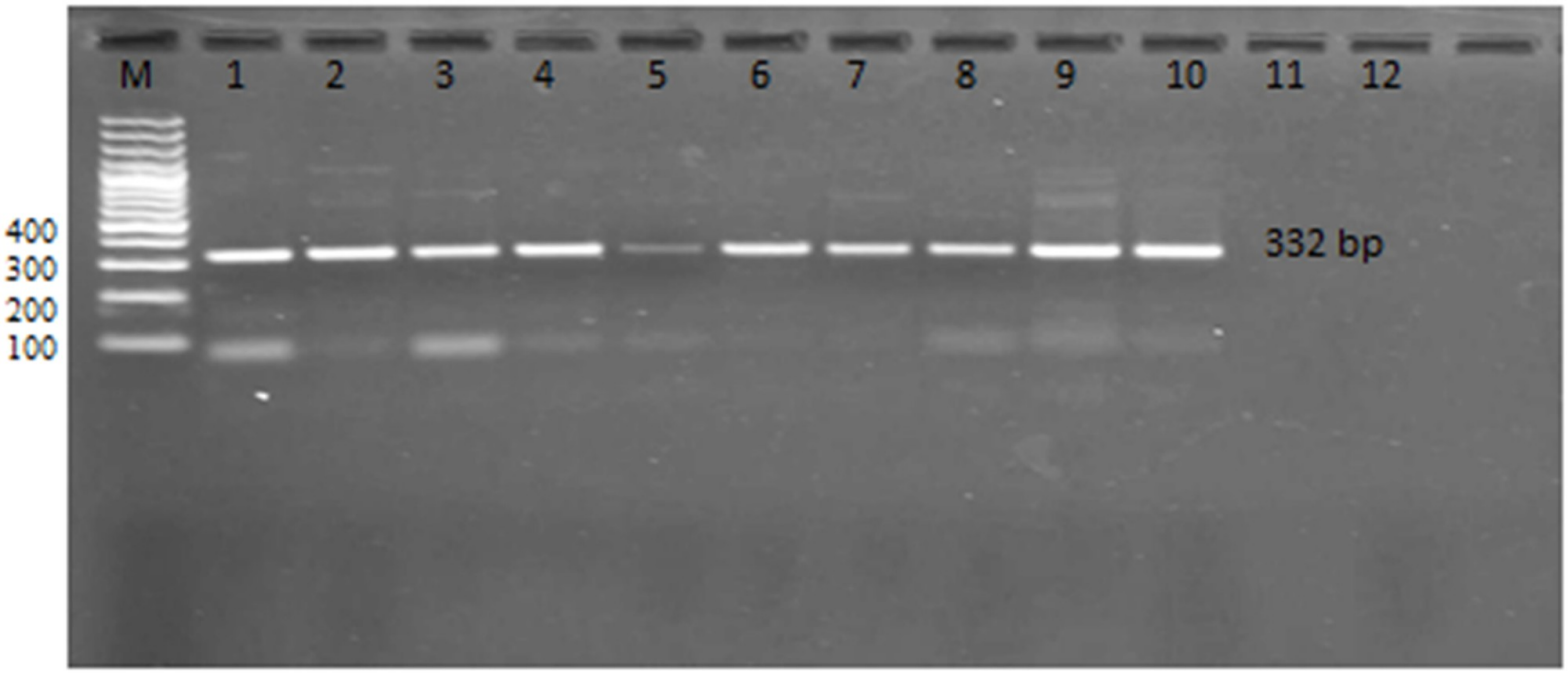
Nested PCR amplification of *Giardia* species-specific primers in 1.5% agarose gel stained with ethidium bromide: Showing nested PCR products of *Giardia* genotype A with positive bands at 332 bp. Lanes (1–10): positive specimens.

**Fig 3:**
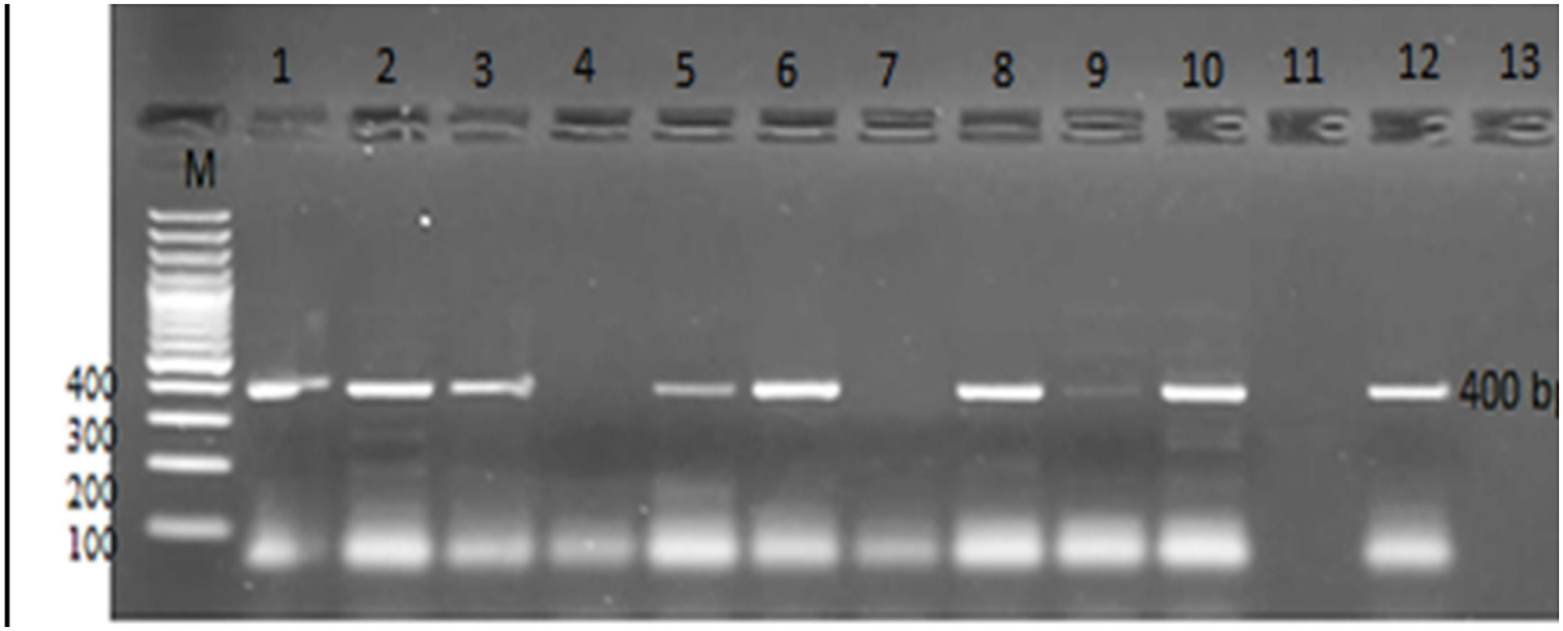
1.5% agarose gel stained with ethidium bromide showing nested PCR amplification of *Giardia* genotype B with positive bands at 400 bp. Lanes (4, 7, 9 & 11): negative genotype B specimens. Lane (M): 100 bp molecular weight ladder.

We identified *G*. *duodenalis* Assemblage A in 45.7% (16/35) of cases using *Giardia* assemblage-specific primers. Assemblage B was identified in 31.4% (11/35) of cases, and mixed infection with A and B assemblages was detected in the remaining cases (22.8%). The zoonotic genotype E was not detected (Figs 2 and 3).

Demographic distribution of *Giardia* genotypes suggested that giardiasis was more prevalent in males (57.1%). However, this association was not statistically significant. Rural communities showed higher rates of infection with assemblage A, which was statistically significant (*p*< 0.05) (17.4%) (Table 5).

**Table 5:** The correlation between different *Giardia* assemblages and patients’ demographic data.

		Assemblage A	Assemblage B	Mixed infection	Total (No./%)	*P*-value
**Gender**	**Males**	**10/20 (50)**	**5/20 (25)**	**5/20 (25)**	**20/35 (57.1)**	**0.674**
	**Females**	**6/15 (40)**	**6/15 (40)**	**3/15 (20)**	**15/35 (42.9)**	
**Residence**	**Rural**	**9/19 (47.4)**	**3/19 (15.8)**	**7/19 (36.8)**	**19/35 (54.3)**	0.035**
	**Urban**	**7/16 (43.75)**	**8/16 (50)**	**1/16 (6.25)**	**16/35 (45.7)**	
**Total**		**16/35 (45.7)**	**11/35 (31.4)**	**8/35 (22.8)**	**35 (100)**	

**p-value: < 0.05 is considered statistically significant.

**Fig 4:**
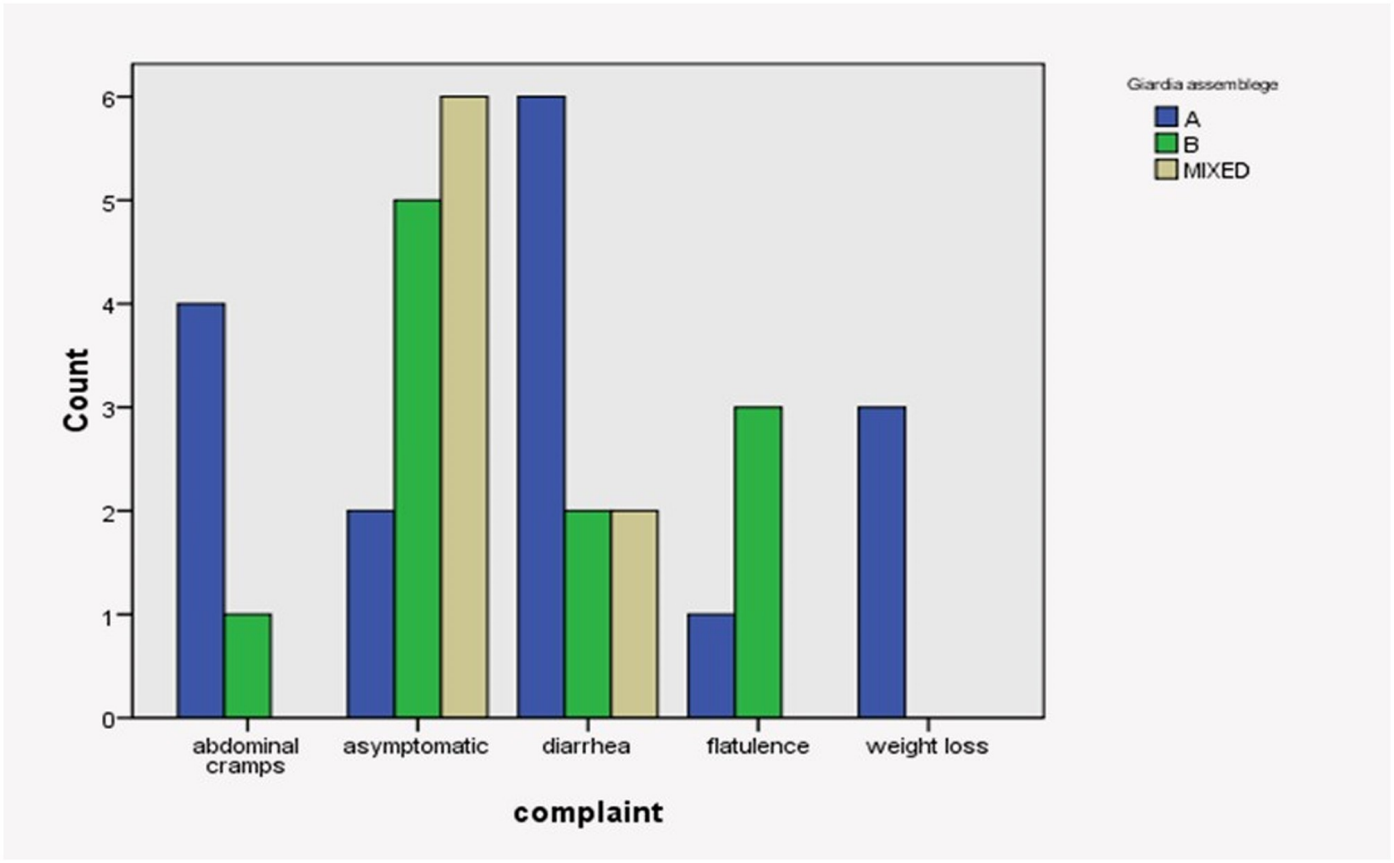
Association between different *Giardia* assemblages with patient symptoms.

Also, Assemblage A was prevalent in symptomatic patients, with 37.5% of patients complained of diarrhea and 25% of abdominal cramps. In contrast, asymptomatic individuals represent 45.4% and 75% of patients with Assemblage B and mixed infections, respectively (Table 6 and Fig 4).

**Table 6:** The association between different *Giardia* assemblages and patients’ symptoms.

Complaint	Assemblage A	Assemblage B	Mixed infection	Total	*P*-value
**Abdominal cramps**	**4/16 (25)**	**1/11 (9.1)**	**0/8 (0)**	**5/35 (14.3)**	0.033**
**Diarrhea**	**6/16 (37.5)**	**2/11 (18.2)**	**2/8 (25)**	**10/35 (28.6)**	
**Flatulence**	**1/16 (6.25)**	**3/11 (27.3)**	**0/8 (0)**	**4/35 (11.4)**	
**Weight loss**	**3/16 (18.75)**	**0/11 (0)**	**0/8 (0)**	**3/35 (8.6)**	
**Asymptomatic**	**2/16 (12.5)**	**5/11 (45.4)**	**6/8 (75)**	**13/35 (37.1)**	
**Total**	**16/35 (45.7)**	**11/35 (31.4)**	**8/35 (22.8)**	**35 (100)**	

**p-value: < 0.05 is considered statistically significant.

## Discussion

Giardiasis is a primary cause of protozoal infection and human diarrhea worldwide. Children are a relatively high-risk group for giardiasis [24, 33]

In the present study, stool samples were collected from 165 children. Microscopy-based prevalence of giardiasis was 24.2%, which is consistent with previous estimates in Egypt that range from 24.7% to 27.9% [34, 35]. Other studies report lower and higher prevalence rates [36, 37]. These differences may be explained by differences in sample size and variation in environmental conditions of studied regions. The majority of infected children were male (57.5%, 23/40), which is likely due to cultural behaviors. Boys tend to be more active outdoors and may have more opportunities for contact with contaminated water or food sources [38].

Several studies targeted different genetic loci in the *G*. *duodenalis* genome for molecular characterization with variable degrees of sensitivity and specificity. In the present study, we used the *tpi* gene for molecular detection of *G*. *duodenalis* owing to its high genetic heterogenicity and polymorphism [15, 39]. Genomic DNA of *G*. *duodenalis* was identified in 35 of 40 microscopically positive samples (87.5%). False-negative PCR results in *Giardia* detection were previously reported [4, 40–42]. Such results were attributed to factors that may affect the DNA yield, such as the presence of DNA inhibitors in stool samples, sample preservation conditions, and the method or type of DNA extraction kit [43]. Also, variations in amplification conditions, the amplification target gene, and the presence of single-nucleotide polymorphisms, insertion-deletions, and presence of different *Giardia* species may cause false-negative findings [44, 45].

The molecular epidemiology of *G*. *duodenalis* was studied in different parts in the world to clarify possible relationships among genetic diversity of the parasite and clinical presentation, drug resistance, and environmental transmission dynamics [46, 47]. Our study used species-specific primers to characterize *G*. *duodenalis* assemblages isolated from children in Upper Egypt. *Giardia* assemblage groups (A and B) were isolated in the present study (45.7%, 31.4%, respectively). These findings are in agreement with other studies that showed both assemblages are most frequently associated with human giardiasis based on analysis of multiple human isolates from different geographical regions [23].

*Giardia duodenalis* Assemblage A is the prevailing genotype in the current study. This assemblage is primarily linked with zoonotic transmission, while Assemblage B is more coupled with human-to-human transmission [48, 49]. However both assemblages have been isolated from humans and domestic animals as dogs, cats and cattle [50]. So, determination of the source of infection may be difficult especially in absence of sub-assemblage analysis. But in the present study we may explain the prevalence of Assemblage A by the significant association between rural residence of the studied population and their high frequency of Assemblage A in stool samples. Close contact with domestic animals and contamination of public water in rural communities provide the opportunity for transmission from animals to humans [51, 52].

The higher prevalence of Assemblage A over Assemblage B was previously reported in from several countries worldwide [53–55]. Our results are also consistent with results obtained by Egyptian researchers who reported a higher prevalence of Assemblage A among Egyptian patients diagnosed with giardiasis [51, 56, 57].

Controversially, several reports document the predominance of *Giardia* Assemblage B in human patients, in Egypt or worldwide [18, 20, 58–63]. However, others observed that both assemblages showed about the same distribution in infected patients [64, 65]. The discrepancy in the distribution of *Giardia* assemblages in human populations observed across countries and even within the same country remains unexplained [46]. The distribution of *G*. *duodenalis* genotypes is likely more related to social and epidemiological criteria of the studied populations or the zoonotic potential in the transmission of giardiasis rather than to geographical association.

Interestingly, mixed infection with assemblages A and B were found in 22.8% of participants. Likewise, the presence of multiple genotypes in one host was reported in other studies, particularly in developing countries [61, 66, 67]. These findings emphasize the complexity and dynamics of the parasite in the ecosystem and reflect the presence of genetically different *Giardia* cysts with mixed sequences contaminating some of the same water and food sources [68, 69]. Further, the occurrence of infection with multiple genotypes favors continued transmission and increased incidence of mixed genotypes in human giardiasis [70]. Interestingly, use of assemblage-specific primers in the current study allowed detection of co-infection with mixed assemblages. *Giardia* super-infection across populations may occur [17, 31, 71].

The role of the genetic diversity of the parasite and its clinical appearance is a controversial topic [47]. Our study found that *Giardia* Assemblage A was prevalent in symptomatic patients, where diarrhea and abdominal cramps were the most common presenting symptoms (37.5% and 25%, respectively). This result was consistent with reports of several studies [11, 72–75]. Such close associations between *Giardia* assemblage A and diarrhoea have also been demonstrated in molecular epidemiological studies conducted in Turkey [72], Spain [76], and Bangladesh [77]. Conversely, *Giardia* Assemblage B and mixed infections were prevalent among asymptomatic individuals (45.4% and 75% of patients, respectively) which is in agreement with Roointan et al. [78] in southwest Iran. Similar to our results, Haque et al. [77] conducted a large scale case control study in Bangladesh involving 3,646 diarrhoeal patients and documented a strong association between assemblage A and symptomatic infection, and between assemblage B and asymptomatic infection, in 211 children under the age of ten.

Unlike our findings, several studies reached different conclusions. Homan and Mank [25] reported that the prevalent genotype in asymptomatic individuals was *Giardia* Assemblage A, and El-Badry et al. [4] in Egypt and others in different countries reported no substantial association between *Giardia* genotype and clinical presentation [21, 22]. Differences in study design, criteria for selecting study populations, and small sample size may explain contradictory findings that were observed in our study and may limit the scope of this study. Also, the study missed investigating the presence of associated enteric viral and bacterial pathogens which could be a potential confounder of the obtained results inducing clinical manifestations and disguising the true clinical effect of *G*. *duodenalis* infection.

More studies on a large scale population are needed to confirm the association between assemblage and symptomology if any exist. Also, more molecular epidemiological studies on wide geographical regions are crucial to explore parasite genotypes, virulence factors, and environmental sources of infections. Indeed, genotyping studies at the sub-assemblage level are essential to ascertain the occurrence and extent of zoonotic transmission events.

## Conclusion

Giardiasis is still a challenging zoonotic disease prevalent among children in the Assiut governorate, Upper Egypt. Our results reveal the predominance of *Giardia* Assemblage A and the presence of mixed infection in a considerable proportion of the study population. These results suggest the zoonotic transmission of infection in our locality rather than human-to-human transmission. Improved control strategies and social awareness of infection transmission are needed, especially in rural areas. In terms of molecular epidemiology, this study illustrates the usefulness and reliability of simple species-specific primers for genotyping *Giardia* spp. and detection of mixed infections. Also, findings highlight the need for further genetic studies to clarify possible correlations between parasite genetic diversity and clinical symptomatology.

## Supporting information

S1 Raw images (PDF)
