# Supplementary figures and images for: Genotyping of Giardia duodenalis in children in upper Egypt using assemblage- specific PCR technique
Source: PLoS One. 2020 Oct 1;15(10):e0240119. doi: 10.1371/journal.pone.0240119 (PMC7529291; doi:10.1371/journal.pone.0240119)

## Supporting information

### Gel raw images

**Fig S1**

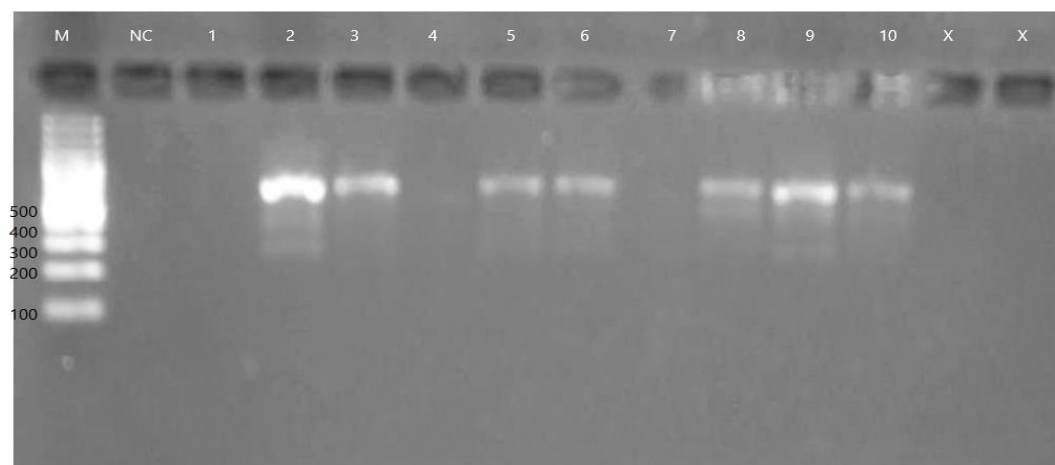

**Fig S2 & 3**

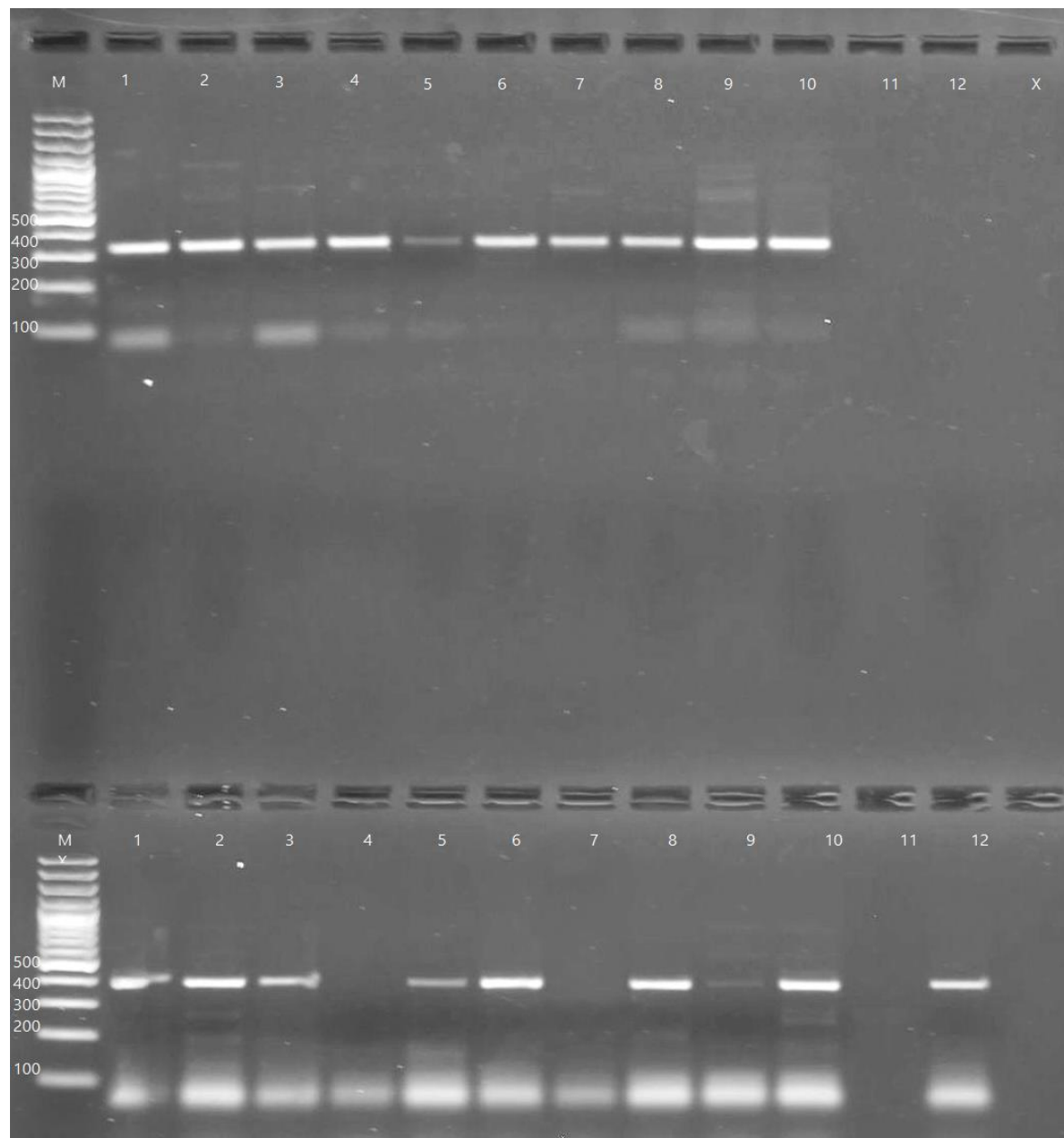

Supplement: S1 Raw images — (PDF) [file pone.0240119.s001.pdf]
